# Automated classification of brain MRI reports using fine-tuned large language models

**DOI:** 10.1007/s00234-024-03427-7

**Published:** 2024-07-12

**Authors:** Jun Kanzawa, Koichiro Yasaka, Nana Fujita, Shin Fujiwara, Osamu Abe

**Affiliations:** Department of Radiology, The University of Tokyo Hospital, Tokyo, Japan

**Keywords:** Brain tumor, Magnetic resonance imaging, Natural language processing, Large language model

## Abstract

**Purpose:**

This study aimed to investigate the efficacy of fine-tuned large language models (LLM) in classifying brain MRI reports into pretreatment, posttreatment, and nontumor cases.

**Methods:**

This retrospective study included 759, 284, and 164 brain MRI reports for training, validation, and test dataset. Radiologists stratified the reports into three groups: nontumor (group 1), posttreatment tumor (group 2), and pretreatment tumor (group 3) cases. A pretrained Bidirectional Encoder Representations from Transformers Japanese model was fine-tuned using the training dataset and evaluated on the validation dataset. The model which demonstrated the highest accuracy on the validation dataset was selected as the final model. Two additional radiologists were involved in classifying reports in the test datasets for the three groups. The model’s performance on test dataset was compared to that of two radiologists.

**Results:**

The fine-tuned LLM attained an overall accuracy of 0.970 (95% CI: 0.930–0.990). The model’s sensitivity for group 1/2/3 was 1.000/0.864/0.978. The model’s specificity for group1/2/3 was 0.991/0.993/0.958. No statistically significant differences were found in terms of accuracy, sensitivity, and specificity between the LLM and human readers (*p* ≥ 0.371). The LLM completed the classification task approximately 20–26-fold faster than the radiologists. The area under the receiver operating characteristic curve for discriminating groups 2 and 3 from group 1 was 0.994 (95% CI: 0.982–1.000) and for discriminating group 3 from groups 1 and 2 was 0.992 (95% CI: 0.982–1.000).

**Conclusion:**

Fine-tuned LLM demonstrated a comparable performance with radiologists in classifying brain MRI reports, while requiring substantially less time.

## Introduction

Brain tumors account for 1.4% of new cancer diagnoses [1]. Despite their rarity, brain tumors pose a remarkable impact on patient outcomes and quality of life [2]. Glioblastomas, the most prevalent type of primary brain cancer arising from glial cells, are almost fatal within 2 years from diagnosis despite comprehensive medical treatment [2]. Moreover, central nervous system cancers are deemed responsible for 7.7 million disability-adjusted life-years globally [2]. This substantial mortality and morbidity require appropriate clinical management and scientific investigation advancement.

MRI has been recognized as the primary imaging modality for brain tumor management, including diagnosis, treatment planning, and monitoring [3]. The widespread adoption of MRI in neuro-oncology has led to a substantial accumulation of radiology report through the years. Effective utilization of this vast data holds great potential in enhancing patient care and supporting research. However, manually organizing extensive data is a time-consuming task, making automation desirable. The development of an automated system for extracting untreated brain tumor reports may serve as an alert mechanism, preventing clinicians from overlooking unexpected brain tumors and promoting timely evaluations. Additionally, it would facilitate research by enabling the efficient creation of patient cohorts from a large number of cases. However, radiology reports in free text render automated analysis difficult [4].

In recent years, the employment of natural language processing (NLP) in the medical field has attracted considerable attention. NLP is a field of artificial intelligence that underscores on the interaction between computers and human language [5]. The advent of large language models (LLM) has revolutionized the NLP performance, leading to widespread research into its clinical applications [6]. LLMs have been applied to numerous radiological tasks, such as automating imaging protocol selection [7], generating report impressions from important findings [8], listing differential diagnosis according to the imaging pattern [9], and generating radiology report from short key words [10]. A Japanese Bidirectional Encoder Representations from Transformers (BERT) model showed promising performance in detecting actionable radiology reports with an area under the receiver operating characteristic curve (AUC) of 0.9516 [11].

LLM can be further trained on radiology-specific dataset, which is referred to as fine tuning. This approach has the potential to improve the model’s performance on radiology-related tasks while decreasing hallucinations [6]. For example, transformer-based language models fine-tuned to radiology tasks performed better than baseline models in abnormal sentence classification, report coding, and report summarization [12].

Although general-purpose, large-scale LLMs such as GPT-4 have demonstrated considerable performance on various radiological tasks [6], these models might pose various issues, such as the requirement of external data transmission. Fine-tuning a smaller, locally hosted LLM is a promising approach to address these issues. This approach allows the model to be downloaded and run on a local environment, protecting the privacy of patients. Furthermore, using a smaller model reduces computational costs, making it feasible to run without extensive resources.

While there have been many reports on the application of LLM in clinical settings, literature on the specific use of LLM for automated brain tumor report classification is lacking. In this study, we aimed to investigate the efficacy of utilizing fine-tuned LLM to properly classify reports of brain tumors for pretreatment, posttreatment, and nontumor cases. We hypothesize that fine-tuned LLM can considerably reduce the time required to complete this complex task while maintaining high accuracy.

## Materials and methods

This retrospective study received approval from our Institutional Review Board, which waived the requirement for obtaining written informed consent from patients owing to the retrospective design of this study.

### Datasets

Picture Archiving and Communication System of our hospital was searched for eligible cases. Radiology reports for brain MRI examination were collated for training, validation, and test dataset. We also searched the teaching file system of our hospital, an archive of educational cases for residents, and associated MRI reports for each case were added to datasets to supplement the lack of pretreatment cases. The composition of each dataset was as follows.

* Training dataset: Brain MRI reports from April 2, 2019 to April 8, 2019 and April 2, 2018 to April 4, 2018 and pretreatment tumor reports for cases from the teaching file system of 2018 and 2019.

* Validation dataset: Brain MRI reports from April 2, 2020 to April 7, 2020 and pretreatment tumor reports for cases from the teaching file system of 2020.

* Test dataset: Brain MRI reports from April 5, 2021 and pretreatment tumor reports for cases from the teaching file system of 2021.

Reports that did not provide enough information to determine whether the tumors were pretreatment or posttreatment were excluded. The reports were stored in CSV format. All reports were mainly written in Japanese, with the diagnosis section containing medical terms in English.

### Reference standard

The clinical indication and imaging diagnosis of each radiological report was reviewed by a radiologist with 2 years of experience in diagnostic imaging and were categorized into three groups: nontumor cases (group 1), posttreatment brain tumor cases (group 2), and pretreatment brain tumor cases (group 3). This categorization was verified by a radiologist with 13 years of imaging experience.

### Fine tuning of the pretrained LLM

This study utilized the pretrained Japanese BERT model (https://huggingface.co/cl-tohoku/bert-base-japanese), version 3. This base-sized model consists of 12 layers, 768 hidden state dimensions, and 12 attention heads, with approximately 110 million parameters in total. The model employs subword tokenization and was pretrained using Japanese Wikipedia data as of September 1, 2019. Fine-tuning was conducted using Python 3.10.13 (https://www.python.org/) and Transformers library 4.35.2 (https://huggingface.co/) on a workstation with a Core™ i9-12900 F central processing unit, an NVIDIA GeForce RTX 3090 graphic processing unit, and 128 GB of random access memory. The Transformers library’s AutoModelForSequenceClassification class method was employed to configure the model for categorizing reports into three groups according to logits. The training session was repeated 15 times with the same hyperparameters and training data to account for the inherent randomness in the fine-tuning process. Each session consisted of 10 epochs to optimize the model’s performance. The number of epochs for the fine-tuning process was determined empirically based on the model’s performance on the training and validation datasets. In each session, the model was fine-tuned via the training dataset and its performance was assessed on the validation dataset. Other hyperparameters were set to default values of the Transformers library. The performance of the model before fine-tuning was also evaluated on the validation dataset. The code used for fine tuning is available upon a reasonable request.

### Test phase of the fine-tuned LLM

The model which showed the highest performance on the validation dataset among 15 sessions was selected as the final model. This model’s performance was further assessed using the independent test dataset. Additionally, to compare the performance of the developed model with that of radiologists, two radiologists (6 and 1 years of imaging experience) manually classified the test dataset reports into three groups. The classified group data and time necessary for completion were recorded.

### Statistical analyses

For the statistical analyses, the R version 4.3.1 (https://www.r-project.org/) was used. The McNemar test was employed to compare the sensitivity, specificity, and accuracy in the test dataset between the fine-tuned LLM and the readers. The receiver operating characteristic (ROC) analysis was performed to evaluate fine-tuned LLM’s diagnostic performance in differentiating groups 2 and 3 from group 1 and group 3 from groups 1 and 2 based on the logit-derived probability with calculating the AUC. Statistical significance was set at a *p*-value of < 0.050.

## Results

### Datasets

Table 1 presents demographic details and group distribution of each dataset. Table 2 summarizes the tumor type frequency presented as a primary diagnosis in the report. The training, validation, and test dataset contained 221/82/456, 89/44/151, and 49/22/93 reports for groups 1/2/3 respectively. The mean age of patients was 53.2 ± 21.3, 55.3 ± 21.2, and 53.5 ± 19.1 years in the training, validation, and test dataset, respectively. The sex distribution was 373 men/386 women in the training dataset, 132 men/152 women in the validation dataset, and 77 men/87 women in the test dataset.

### Model selection

Table 3 summarizes the performance of models from each training session on the validation dataset. The model from the 4th training session demonstrated the highest accuracy and was selected as the final model.

### Performance of the fine-tuned LLM and radiologists on the test dataset

Table 4 demonstrates a confusion matrix for the reference standard and prediction data. Table 5 shows the performance of the fine-tuned LLM and radiologists in classifying the test dataset.

The fine-tuned LLM attained an overall accuracy of 0.970 (95% CI: 0.930–0.990), which was comparable to readers 1 and 2.

The LLM sensitivity for groups 1 (1.000, 95% CI: 0.927–1.000) and 3 (0.978, 95% CI: 0.924–0.997) was comparable to both readers. For group 2, the LLM sensitivity was 0.864 (95% CI: 0.651–0.971), which tended to be slightly lower than human readers.

The LLM specificity ranged from 0.958 (95% CI: 0.881–0.991) in group 3 to 0.993 (95% CI: 0.961–1.000) in group 2. These specificities were similar to those of the human readers.

No statistically significant differences were found in terms of accuracy, sensitivity, and specificity between the LLM and readers for any group.

**Fig. 1:**
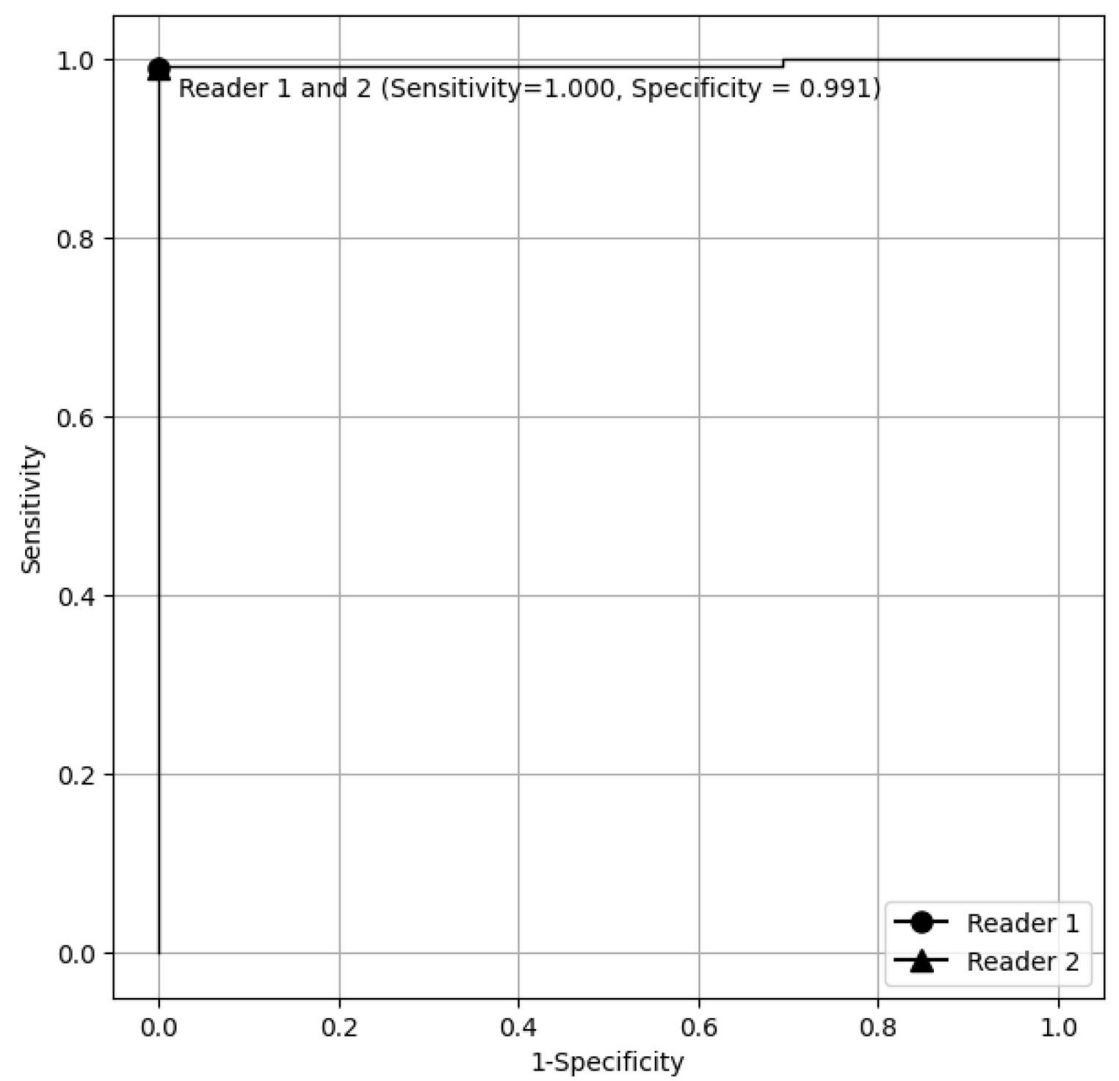
Receiver operating characteristic curve for the fine-tuned large language model in discriminating groups 2 and 3 (reports with brain tumor) from group 1 (reports without brain tumor) in the test dataset. The result of the two radiologists, which was identical, is plotted on the graph

**Fig. 2:**
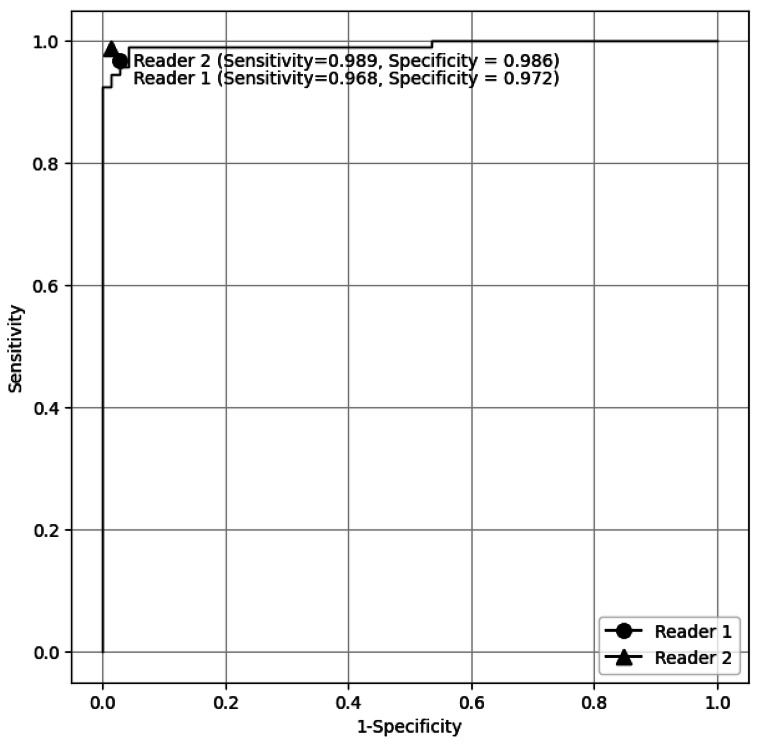
Receiver operating characteristic curve for the fine-tuned large language model in discriminating group 3 (reports with pretreatment brain tumor) from groups 1 and 2 (reports with posttreatment brain tumor and reports without brain tumor) in the test dataset. The result of two radiologists is illustrated on the graph

Figure 1 demonstrates the ROC curve for fine-tuned LLM in discriminating groups 2 and 3 (reports with brain tumor) from group 1 (reports without brain tumor) in the test dataset. The AUC was 0.994 (95% CI: 0.982–1.000). Figure 2 shows the ROC curve for the fine-tuned LLM in discriminating group 3 (reports with pretreatment brain tumor) from group 1 and 2 (reports with posttreatment brain tumor and reports without brain tumor) in the test dataset. The AUC was 0.992 (95% CI: 0.982–1.000).

The fine-tuned LLM required substantially less time to classify the test dataset compared to human readers. The LLM completed the classification task in 57 s, which was approximately 26-fold faster than reader 1 (1487 s) and 20-fold faster than reader 2 (1131 s).

## Discussion

Our study reveals that fine-tuned LLM can stratify brain MRI reports into no brain tumors, posttreatment brain tumors, and pretreatment brain tumors with high accuracy, sensitivity, and specificity, comparable to human readers. LLM required remarkably less time to complete the classification task.

The ability of LLM to efficiently extract pretreatment cases (group 3) indicates its potential utility in clinical and research purposes. It can alert clinicians, promptly reminding them of the unexpected presence of brain tumors. This can prevent clinicians from overlooking cases that require further investigation while checking a large number of reports and facilitate timely assessment. It can also be utilized to create cohorts for brain tumor research by helping the identification of relevant cases from the vast radiology report archives. LLM also conducted well in identifying reports without brain tumors (group 1), implying that LLM is also useful in creating control groups in brain tumor studies.

LLM demonstrated a lower performance than human readers for posttreatment brain tumor reports (group 2). In clinical and research practice, this issue may not be a critical weak point, as patients with tumors tend to be closely followed up both before and after treatment. This close follow-up renders the identification of posttreatment cases easier once initial reports are identified.

In previous research, Kehl et al. employed deep NLP techniques to extract information from radiology reports, such as existence of tumors and interval change of lesions [13]. They showed that a deep-learning model demonstrated high AUC over 0.90 for the detection of any cancer, worsening/progression, and improvement/response. As for the detection of brain/spine lesions, the AUC of their model was 0.97. Nakamura et al. demonstrated that a Japanese BERT model showed a high AUC of 0.9516 for detecting actionable radiology reports [11]. Our result corroborates with these previous reports, confirming the promising potential of NLP to efficiently treat information in radiology reports.

Our study is unique in its focus on the use of fine-tuned LLM for report classification task. A prior study reported that LLM, particularly those pretrained on biomedical data, exhibits excellent performance on a wide range of NLP tasks with limited requirements for task-specific architectural modifications [14]. Given the optimal performance of fine-tuned LLM in classifying brain MRI reports, we propose that our approach can be extended to a variety of other radiology tasks. This study is also notable for demonstrating that even small, locally available LLMs can achieve sufficient performance through fine-tuning. This approach ensures privacy of patients by keeping sensitive data within a secure local environment. Furthermore, the low computational requirements of smaller LLMs make them feasible to implement in clinical settings.

This study had some limitations. First, this retrospective study was performed at a single institution. As the format of imaging reports may vary between institutions, model adjustments may be necessary to generalize our results to other hospitals. In addition, since the data was collected from a university hospital, the disease distribution may be unique compared to other facilities. Second, our study underscored on the existence of brain tumors; thus, the ability to identify other lesions was not evaluated. Finally, the LLM pretrained with Japanese data was utilized for the fine tuning. Our results may not necessarily apply to other languages. Future studies are required to validate our approach using datasets from various institutions, languages, and disease distributions.

In conclusion, a fine-tuned LLM demonstrated comparable performance with radiologists in classifying brain MRI reports, while requiring substantially less time. Integrating these findings into clinical workflows can contribute to clinicians by preventing them from overlooking brain tumors identified in reports and helping them identify relevant cases for research purposes from a large number of cases.

**Table 1:** Demographic details and group distribution of each dataset

	Training	Validation	Test
Number of reports	759	284	164
Age (mean ± SD)	53.2 ± 21.3	55.3 ± 21.2	53.5 ± 19.1
Sex (male/female)	373 / 386	132 / 152	77 / 87
Number of reports in each group			
1: reports without brain tumor	221	89	49
2: reports with posttreatment brain tumor	82	44	22
3: reports with pretreatment brain tumor	456	151	93

**Table 2:** Frequency of tumor types presented as a primary diagnosis

Tumor type	Training	Validation	Test
Glioma	91	80	42
Meningioma	84	15	10
Schwannoma	75	13	5
Pituitary tumor	59	11	6
Chordoma/chondrosarcoma	58	15	3
Glioneural tumor	51	13	4
Hemangioblastoma	33	0	16
Metastasis	24	12	13
Ependymoma/subependymoma	19	6	4
Epidermoid	9	0	1
Lymphoma	4	11	0
Other	31	19	11

**Table 3:** Model’s performance on the validation dataset

Model	Accuracy	Sensitivity 1	Sensitivity 2	Sensitivity 3	Model	Accuracy	Sensitivity 1	Sensitivity 2	Sensitivity 3
0	0.419	0.000	0.068	0.768	8	0.944	0.933	0.854	0.973
1	0.954	0.933	0.909	0.980	9	0.930	0.910	0.795	0.980
2	0.937	0.888	0.909	0.974	10	0.947	0.933	0.886	0.974
3	0.940	0.888	0.864	0.993	11	0.947	0.910	0.864	0.993
4	0.958	0.955	0.886	0.980	12	0.947	0.899	0.886	0.993
5	0.933	0.921	0.818	0.974	13	0.947	0.955	0.818	0.980
6	0.933	0.910	0.864	0.967	14	0.944	0.888	0.886	0.993
7	0.940	0.944	0.795	0.980	15	0.933	0.876	0.909	0.974

Sensitivity 1, 2, and 3 stands for sensitivity for groups 1, 2, and 3, respectively

Model 0 stands for the model before fine-tuning

Model 1 to 15 stand for the model from each 15 training sessions

**Table 4:** Confusion matrix for the reference standard vs. prediction data in the test dataset

	Reference standard		
	Group 1 (*n* = 49)	Group 2 (*n* = 22)	Group 3 (*n* = 93)
Large language model			
Group 1 (*n* = 49)	49	0	1
Group 2 (*n* = 20)	0	19	1
Group 3 (*n* = 94)	0	3	91
Reader 1			
Group 1 (*n* = 50)	49	0	1
Group 2 (*n* = 22)	0	20	2
Group 3 (*n* = 92)	0	2	90
Reader 2			
Group 1 (*n* = 50)	49	0	1
Group 2 (*n* = 21)	0	21	0
Group 3 (*n* = 93)	0	1	92

**Table 5:** Performance comparison of fine-tuned large language model and radiologists in classifying the test dataset

	Fine-tuned LLM	Reader 1		Reader 2	
		score	Comparison	score	Comparison
Accuracy	0.970 (0.930–0.990)	0.970 (0.930–0.990)	1.000	0.988 (0.957–0.999)	0.371
Sensitivity for each group					
Group 1	1.000 (0.927–1.000)	1.000 (0.927–1.000)	-	1.000 (0.927–1.000)	-
Group 2	0.864 (0.651–0.971)	0.909 (0.708–0.989)	1.000	0.955 (0.772–0.999)	0.480
Group 3	0.978 (0.924–0.997)	0.968 (0.909–0.993)	1.000	0.989 (0.942–1.000)	1.000
Specificity for each group					
Group 1	0.991 (0.953–1.000)	0.991 (0.953–1.000)	1.000	0.991 (0.953–1.000)	1.000
Group 2	0.993 (0.961–1.000)	0.986 (0.95–0.998)	1.000	1.000 (0.974–1.000)	-
Group 3	0.958 (0.881–0.991)	0.972 (0.902–0.997)	1.000	0.986 (0.924–1.000)	0.480
Time required (s)	57	1487		1131	

Comparisons between fine-tuned LLM vs. readers were conducted with the McNemar test. LLM = large language model

## Data Availability

The datasets and code used in this study are available from the corresponding author on reasonable request.
